# Coarse-to-Fine Adaptive People Detection for Video Sequences by Maximizing Mutual Information †

**DOI:** 10.3390/s19010004

**Published:** 2018-12-20

**Authors:** Álvaro García-Martín, Juan C. SanMiguel, José M. Martínez

**Affiliations:** Video Processing and Understanding Lab (VPULab), Universidad Autónoma de Madrid, 28049 Madrid, Spain; juancarlos.sanmiguel@uam.es (J.C.S.); josem.martinez@uam.es (J.M.M.)

**Keywords:** people detection, detector adaptation, pair-wise correlation, thresholds, entropy, coarse-to-fine adaptation

## Abstract

Applying people detectors to unseen data is challenging since patterns distributions, such as viewpoints, motion, poses, backgrounds, occlusions and people sizes, may significantly differ from the ones of the training dataset. In this paper, we propose a coarse-to-fine framework to adapt frame by frame people detectors during runtime classification, without requiring any additional manually labeled ground truth apart from the offline training of the detection model. Such adaptation make use of multiple detectors mutual information, i.e., similarities and dissimilarities of detectors estimated and agreed by pair-wise correlating their outputs. Globally, the proposed adaptation discriminates between relevant instants in a video sequence, i.e., identifies the representative frames for an adaptation of the system. Locally, the proposed adaptation identifies the best configuration (i.e., detection threshold) of each detector under analysis, maximizing the mutual information to obtain the detection threshold of each detector. The proposed coarse-to-fine approach does not require training the detectors for each new scenario and uses standard people detector outputs, i.e., bounding boxes. The experimental results demonstrate that the proposed approach outperforms state-of-the-art detectors whose optimal threshold configurations are previously determined and fixed from offline training data.

## 1. Introduction

Automatic people detection in video sequences is one of the most relevant problems in computer vision, which is essential in many applications such as for video-surveillance, human–computer interaction and mobile robotics. Although generic object detection is maturing very rapidly thanks to the recent widespread use of deep learning [1,2], many challenges still exist for the specific case of detecting people. Video and images of people exhibit a great variation of viewpoints, motion, poses, backgrounds, occlusions, sizes and body-part deformations [3]. Detection performance has a strong dependency on the training data used to build detectors [4] and, therefore, accuracy drops are expected when training and testing data have different patterns [5]. Moreover, people detectors often have many parameters, which are heuristically or experimentally set according to training data. Such parameter setting strategy may have limitations when applied to other data different from the training one.

The adaptation of people detectors is therefore desired to successfully apply such detectors to unseen data [6]. This adaptation can be approached as best algorithm selection [7,8], domain adaptation for learning scene-specific detectors [9], data augmentation for the video-surveillance domain [10] and unsupervised feature learning [3,4]. However, these approaches imply retraining models for the new target domain, which may not be possible in certain applications such as real-time video-surveillance where data may not be available in advance. Alternatively, one may adapt detectors for testing time without changing any model by combining multiple features [11], embedding detection within a multi-class Bayesian classification [5], designing cascades of heterogeneous detectors [12] or coupling detection and tracking [13,14]. However, these approaches impose restrictions on the employed detectors (e.g., high precision and low recall [14]) or require the use of tracking [13,14].

**Figure 1:**
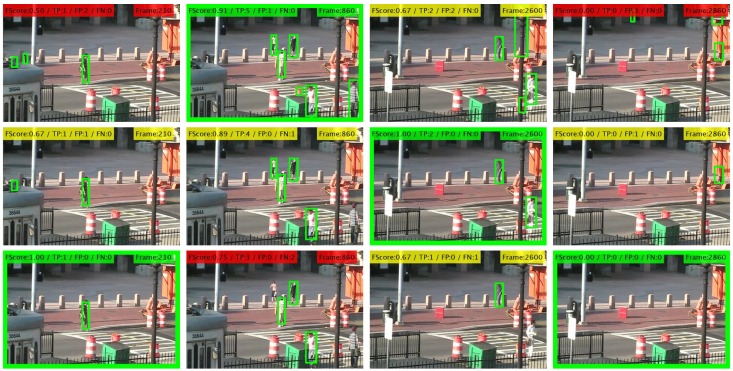
People detection results for Faster R-CNN [1] of detectors (sequence *tramstop*, http://www-vpu.eps.uam.es/PDbm), in terms of FScore, true positive detections (TP), false positive detections (FP) and false negative detections (FN). Each row corresponds to applying a detection threshold with values 0.24 (Row 1), 0.5 (Row 2) and 0.75 (Row 3). Finding an optimal threshold (framed in green) for all cases is challenging due to the variability of viewpoints, people sizes and occlusions.

To overcome the above-mentioned shortcomings, in this paper, we propose a coarse-to-fine framework to adapt the configuration of people detectors during testing time. In particular, we focus on the thresholding stage that determines the detector output (i.e., bounding boxes), being quite popular among a wide variety of recent detectors and having a strong impact on detector’s performance (see examples in Figure 1). We employ multiple detectors to simultaneously find their optimal threshold values within an optimization framework based on their mutual information [15]. Our proposal explores multiple thresholding hypotheses for all employed detectors and exploits pair-wise correlations between their outputs within a coarse-to-fine adaptation strategy. First, a coarse stage employs correlation entropy to identify which frames of the video sequence contain people and therefore enables speeding up the detection process by avoiding analyzing frames without people. Second, a fine adaptation stage is performed for frames where people are present by optimally selecting the detection threshold for each detector. Such selection is performed for each detector by accumulating all pair-wise comparisons with other detectors. Finally, we obtain the output of each detector by applying the obtained threshold value. The proposed framework only requires threshold-based detectors with an output in the form of bounding boxes. Therefore, it can be applied to many recent approaches, as demonstrated by the experimental results, which show that adapting sets of people detectors (from two to six) outperforms individual detectors tuned to obtain maximum performance (i.e., whose threshold is trained offline and fixed in advance). Preliminary results are published in [15].

The remainder of the paper is structured as follows. Section 2 describes the related work. Section 3 describes the proposed coarse-to-fine adaptation framework based on cross-correlations. Section 4 presents the experiments. Finally, Section 5 concludes this paper.

## 2. State of the Art

Adapting pedestrian detectors to specific scenes is frequently termed as *domain adaptation* where the original training dataset (i.e., *source domain*) is fully annotated. Existing approaches adapt such detectors to unseen data (i.e., *target domain*) which can be focused on features or models [16].

*Feature-based approaches* aim to transform feature spaces between the source and target domains, and then apply a classifier. Early approaches annotate data in the target domain to define a grid classifier from scratch [17]. Albeit effective, such annotation is time-demanding, several data samples are needed and therefore difficult to perform for other domains. Most of recent feature-based approaches focus on *transfer learning* where the knowledge from source domains is extended to semantically-similar categories of the target domain by retraining models with few data annotations. Transfer-learning can use bounding boxes from both the source and target domain such as the learning of discriminative models using CNNs and data augmentation [10] and the transfer of shared source-target attributes by feature selection where data distributions of the domains are similar [18]. Moreover, approaches can also assume the absence of annotations for the target domain and, therefore, perform an online self-learning process by determining which samples to select. For example, such selection can use super-pixel region clustering [19], Gaussian regression within a hierarchical adaptive SVM [16], confidence scores within a deep model [3], background modeling [20] and multiple contextual cues [4]. Other strategies may also be applied by weighting the source data to match the distribution of the object categories in the target domain before re-training [4], by propagating labels between frames for good positive instances [20] and by integrating classifiers at image and instance level to maintain semantic consistency between two domains [21]. Image level aims to determine whether source or target domains are analyzed, whereas instance level classifier is focused on the feature maps. Finally, transfer learning using synthetic data has recently been proposed [22,23]. However, training complex models still presents challenges due to the visual mismatch with real data [20].

*Model-based approaches* focus on adapting the parameters of the classifiers or the strategy applied. For example, in [5], a Bayes-based multi-class classifier is adapted by computing the proportion of objects in the target domain during runtime. Such adaptation may focus on correcting detection errors by spatiotemporal filtering [24]. Other approaches make *use of context* such as for building a partial belief about the current scene to only execute certain classifiers [25], for applying specific combinations of part-based models based on spatial object information [26] and for modulating object proposals (class prior probabilities) with semantic knowledge [27]. Model-based approaches may also combine different models by learning the weights of predictions for different sensor modalities in an online manner [11], by applying a cascade of detectors designed to combine the confidence of heterogeneous detectors [12], and by selecting automatically the most suitable model for visible or non-visible light images [28]. Another approach focuses on automatically learning classifiers on the target domain without annotated data, which are later evaluated in the source domain with labeled data and finally top-performing classifiers are selected as the most reliable for the target domain [29]. Moreover, model-based approaches may perform *detector ranking* by estimating the similarity between both domains in some feature space to design a cost function for selecting the best algorithm in each situation or domain [7]. Therefore, detector ranking can be efficiently learned for different target domain subsets [8] but requires full annotation of source and target domain. Similar to feature-based approaches, model-based detector adaptation may be achieved by *coupling detection and tracking* for online retraining single [13] or multiple [14] detectors without annotated data. However, these approaches share the limitations of transfer learning (detector re-training), impose restrictions on the employed detectors (e.g., high precision and low recall [14]) or require the use of tracking which is may lead to unstable results [13,14].

Table 1 compares the proposed and reviewed approaches. As we can observe, the proposed approach avoids re-training detectors, unlike many model-based and feature-based approaches based on transfer learning, which often require an offline training stage before the final application to the target domain. Instead of selecting accurate samples for re-training, we leverage results from multiple and possibly independent people detectors assuming that their errors are diverse. The detection threshold of each detector is adjusted according to similarities to other employed detectors. Moreover, our proposal applies self-learning in an online fashion without requiring annotated data for the target domain, unlike those in [26,27] and also without requiring a prior analysis of the target domain features [7,8]. Additionally, the proposed approach employs standard outputs of people detectors (i.e., bounding boxes) so it can be applied to a wide variety of existing approaches, unlike other approaches restricted to CNNs [10], Faster R-CNN [21], and SVMs [16,18] or to being coupled with other detectors [11] and trackers [13]. Finally, the proposed approach is applied to video sequences, unlike most of those in the literature, which are focused on image-level classification. Such application to video may determine when and where adaptation might improve performance, and therefore adjust the computational complexity to the particular details of each video sequence.

## 3. Detector Adaptation Framework

**Figure 2:**
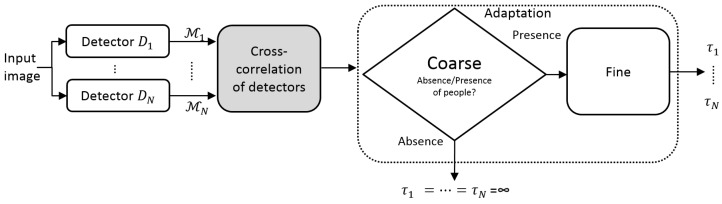
Overview of coarse-to-fine adaptation system.

We propose a coarse-to-fine framework to improve detector’s performance at runtime classification by adapting the configuration of each detector employed (see Figure 2). This proposal is inspired by the *maximization of mutual information* strategy where classifiers are combined assuming that their errors are complementary, being successfully applied for example to detect shadows [30] and skin [31]. We extend such maximization framework to people detection by introducing pair-wise detector correlation and by adapting online their configuration. Note that we are not re-training detectors at prediction time, which may require data not available in real applications or highly-accurate detectors, and may imply high latency [5], i.e., a minimum number of frames to compute accurate decisions over time. Instead, we consider generic threshold-based detectors pre-trained on standard datasets, thus making this proposal applicable to a wide variety of detectors.

Assuming a set of *N* people detectors ${\left\{D_{n}\right\}}_{n=1}^{N}$ applied to an image, each detector $D_{n}$ obtains a confidence map $M_{n}$ describing the people likelihood for each spatial location (x,y) and scale *s* in the image. Then, detection candidates are obtained by thresholding this map:

(1) $$T_{n}\left(x,y,s\right)=\left\{\begin{array}{ccc} 1 & if & M_{n}\left(x,y,s\right)>\tau_{n}\\ 0 & otherwise \end{array},\right.$$

where $T_{n}\left(x,y,s\right)=\left\{0,1\right\}$ and $\tau_{n}$ is the detection threshold whose value is heuristically set based on the confidence map. These candidates are later combined across scales and can be post-processed by a variety of techniques such as non-maximum suppression [32] and background-people segmentation [33]. The final result for each detector is a set $B_{n}^{\tau_{n}}={\left\{b_{k}\right\}}_{k=1}^{k=K^{\tau_{n}}}$ with $K^{\tau_{n}}$ detections (i.e., bounding boxes) representing the output of the detector $D_{n}$ where each detection $b_{k}$ (i.e., bounding box) is described by its position (x,y) and dimensions (w,h). A key parameter in this procedure is the detection threshold $\tau_{n}$, which determines the number of detection candidates. Low (high) values of $\tau_{n}$ generate several (few) detections increasing the false (true) positive rate: three examples of $\tau_{n}$ are shown in Figure 1. We propose to adapt such detection threshold to the image context by exploring similarities with the other detectors.

We compare the output of detectors to obtain a set of pair-wise correlation scores (*cross-correlation of detectors* in Figure 2), which measures the output similarity. This stage is extended in Section 3.1.

We analyze this similarity at two different levels. First, we propose a *coarse* analysis to determine relevant frames in a video sequence, where people are present. Second, a *fine* analysis is applied in those selected frames to adapt the detection system, i.e., adjust the detection thresholds.

### 3.1. Cross-Correlation of Detectors

**Figure 3:**
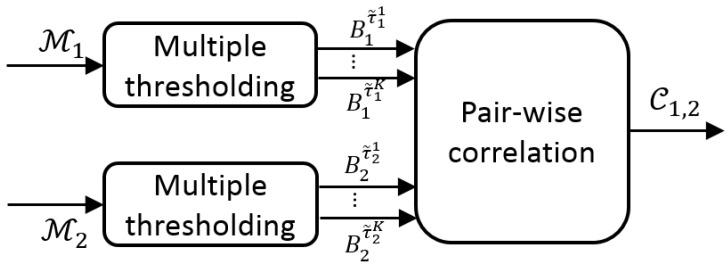
Cross correlation of detectors overview.

Firstly, we explore the decision space to determine each detector output by applying multiple thresholds. Then, we correlate these multiple outputs for each pair of detectors ($D_{n}$ and $D_{m}$) to obtain a correlation map $C_{n,m}$ which measures the output similarity (see Figure 3).

#### 3.1.1. Multiple Thresholding

**Figure 4:**
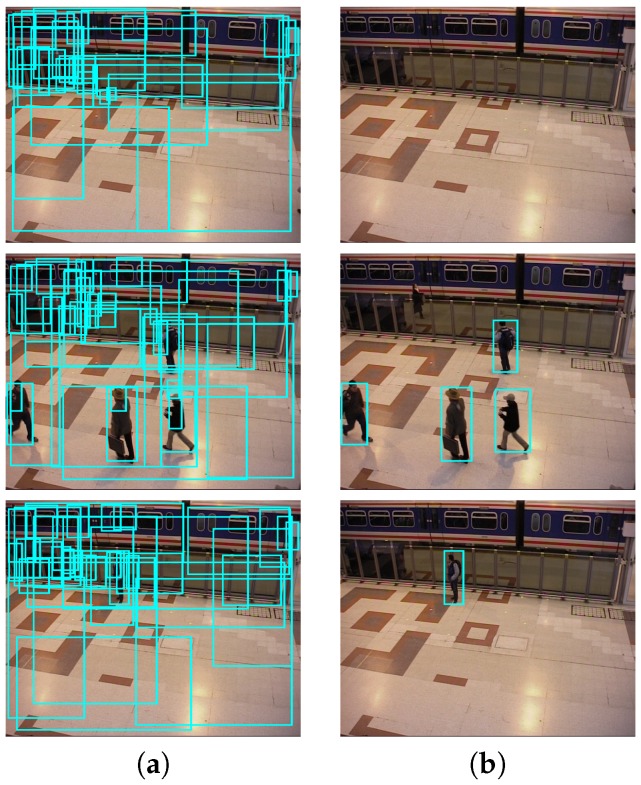
Multiple thresholding examples with Faster R-CNN detector [1]. Three examples (rows) are shown where columns are obtained bounding boxes for thresholds (**a**) ${\tilde{\tau }}_{1}^{1}=0$ and (**b**) ${\tilde{\tau }}_{1}^{52}=0.85$ from the full set ${\left\{{\tilde{\tau }}_{n}^{j}\right\}}_{j=1}^{j=L=60}$.

To explore the possible detector outputs, we define a set of *L* thresholds ${\left\{{\tilde{\tau }}_{n}^{j}\right\}}_{j=1}^{j=L}$ for each detector $D_{n}$ whose values are determined by considering *L* levels between the extreme values of the confidence map $M_{n}$ (i.e., minimum and maximum). Then, we perform thresholding with multiple values ${\tilde{\tau }}_{n}^{j}$ to obtain a set of outputs as follows:

(2) $$\Omega_{n}=\left\{B_{n}^{{\tilde{\tau }}_{n}^{j}}\right\};1\leq j\leq L,$$

where each output $B_{n}^{{\tilde{\tau }}_{n}^{j}}$ is obtained by applying the threshold ${\tilde{\tau }}_{n}^{j}$ to Equation (1). Note that each detector $D_{n}$ may have different threshold values ${\tilde{\tau }}_{n}^{j}$ adapted to the range of values in $M_{n}\left(x,y,s\right)$. Figure 4 shows three examples (rows) of the possible detector outputs $B_{n}^{{\tilde{\tau }}_{n}^{j}}$ obtained by applying two different thresholds j=1 and j=52 from the full set ${\left\{{\tilde{\tau }}_{n}^{j}\right\}}_{j=1}^{j=L=60}$.

#### 3.1.2. Pair-Wise Correlation

We correlate the *N* detector outputs ${\left\{\Omega_{n}\right\}}_{n=1}^{n=N}$ to estimate their similarity. We compute a correlation map $C_{n,m}$ for each pair of detectors outputs $\Omega_{n}$ and $\Omega_{m}$. Each element is defined as:

(3) $$C_{n,m}\left(i,j\right)=\rho \left(B_{n}^{{\tilde{\tau }}_{n}^{i}},B_{m}^{{\tilde{\tau }}_{m}^{j}}\right),\;\left(i,j\in \{1,\ldots L\}\right)$$

where ρ(·,·) is a function to compute the similarity between the output of detectors. The number of correlation maps $C_{n,m}$ to be computed for N detectors is $\left(\begin{array}{c} N\\ 2 \end{array}\right)=\frac{N!}{2\cdot (N-2)!}$.

We propose computing ρ(·,·) as a one-class classification problem by applying standard evaluation measures. To compare bounding boxes from two outputs, we use three matching criteria [34]: relative distance $dr\in [0,d_{max}]$ (where $d_{max}$ is the image diagonal divided by each $b_{j}$ size), cover co∈[0,1] and spatial overlap ov∈[0,1]. The criterion dr measures the distance between the bounding box centers of $B_{n}^{{\tilde{\tau }}_{n}^{i}}$ and $B_{m}^{{\tilde{\tau }}_{m}^{j}}$ in relation to the size of the bounding boxes in $B_{m}^{{\tilde{\tau }}_{m}^{j}}$. Similar to dr, criteria co and ov employ, respectively, the percentage of spatial bounding box coverage in $B_{m}^{{\tilde{\tau }}_{m}^{j}}$ and the intersection-over-union features. A positive match is considered true if dr≤0.5, co≥0.5 and ov≥0.5, as commonly employed in related works [34], which corresponds to a deviation up to 25% of the true object size. Only one $b_{k}\in B_{n}^{{\tilde{\tau }}_{n}^{i}}$ is accepted as correct by matching $b_{l}\in B_{n}^{{\tilde{\tau }}_{m}^{j}}$ (i.e., true positive), so any additional $b_{k}\in B_{n}^{{\tilde{\tau }}_{n}^{i}}$ on the same bounding box is considered as a false positive. Then, we compute precision and recall measures from the matching results and obtain the FScore as the final similarity measure ρ(·,·) between $B_{n}^{{\tilde{\tau }}_{n}^{i}}$ and $B_{m}^{{\tilde{\tau }}_{m}^{j}}$ as in [35].

Thus, the final correlation map $C_{n,m}$ between two detectors is defined as the FScores *F*:

(4) $$C_{n,m}=\left[\begin{array}{ccc} F\left(B_{n}^{{\tilde{\tau }}_{n}^{1}},B_{m}^{{\tilde{\tau }}_{m}^{1}}\right) & \ldots & F\left(B_{n}^{{\tilde{\tau }}_{n}^{1}},B_{m}^{{\tilde{\tau }}_{m}^{L}}\right)\\ \ldots & F\left(B_{n}^{{\tilde{\tau }}_{n}^{i}},B_{m}^{{\tilde{\tau }}_{m}^{j}}\right) & \ldots \\ F\left(B_{n}^{{\tilde{\tau }}_{n}^{L}},B_{m}^{{\tilde{\tau }}_{m}^{1}}\right) & \ldots & F\left(B_{n}^{{\tilde{\tau }}_{m}^{L}},B_{m}^{{\tilde{\tau }}_{m}^{L}}\right) \end{array}\right],$$

where i,j={1,…,L}.

**Figure 5:**
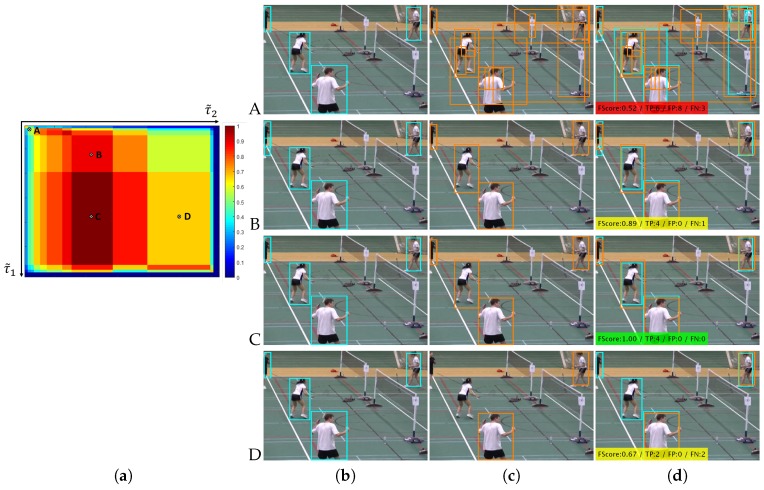
Correlation map example $C_{1,2}$ for two Faster R-CNN detectors [1] using VGG (cyan) and ZF (orange) models (**a**). Four different outputs examples $C_{1,2}\left(i,j\right)$ (rows A, B, C and D) are shown where columns are the corresponding bounding boxes of: (**b**) the VGG detector $B_{1}^{{\tilde{\tau }}_{1}^{i}}$; (**c**) the ZF detector $B_{2}^{{\tilde{\tau }}_{2}^{j}}$; and (**d**) the associated correlation similarity result.

Figure 5 shows one example of correlation map $C_{1,2}$ and four different outputs between two the detectors $C_{1,2}\left(i,j\right)$ (rows A, B, C and D). Example A corresponds to a low threshold value for both detectors (${\tilde{\tau }}_{1}^{i}$ and ${\tilde{\tau }}_{2}^{j}$) and therefore in this case a low FScore similarity F(i,j)=0.52. On the other hand, Example C corresponds to a medium-high threshold value for the first detector ${\tilde{\tau }}_{1}^{i}$ and a low-medium threshold value for the second detector ${\tilde{\tau }}_{2}^{j}$, and therefore in this case a high FScore similarity F(i,j)=1.0.

### 3.2. Coarse Adaptation

**Figure 6:**
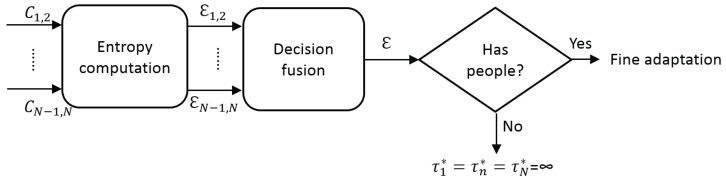
Overview of coarse adaptation.

Assuming that frames without people are not relevant for the adaptation process, we propose to use the correlation map $C_{n,m}$ to determine the relevant frames in a video sequence. In particular, we propose to measure the information entropy as an estimation of the presence of people in every frame (see Figure 6).

Based on the principle of maximization of mutual information, we assume that two independent detectors, albeit designed for the same purpose (to detect persons), in presence of people would be highly correlated when many bounding boxes are matched and, therefore, a high level true positive detections is expected. On the other hand, low correlation values would have few matches and, therefore, imply an increase in the false positive rate or negative detection rate. Note that there is one exception to this assumption when outputs are empty (i.e., $B_{n}^{{\tilde{\tau }}_{n}^{i}}=B_{m}^{{\tilde{\tau }}_{m}^{j}}=\emptyset$) since both outputs are equal and we cannot compute the FScore. To consider this, we avoid this situation by setting the FScore to zero when these sets are empty. However, two independent detectors applied to a frame without the presence of people would have low correlation values for every possible configuration $C_{n,m}$. For that reason, we can assume that those frames with the presence of persons will produce more variable correlation maps $C_{n,m}$ than those without people.

We propose estimating the absence/presence of people using the entropy of the correlation map $C_{n,m}$. Information entropy is defined as the average amount of information produced by a stochastic source of data. The measure of information entropy associated with each possible data value is the negative logarithm of the probability mass function for the value. Entropy is a statistical measure of randomness that can be used to characterize the texture of an input image. In our case, we propose classifying every frame using the entropy over the correlation map $C_{n,m}$ as:

(5) $$E_{n,m}=-\sum_{i,j}C_{n,m}\left(i,j\right)\cdot log\left(C_{n,m}\left(i,j\right)\right),\;\left(i,j\in \{1,\ldots L\}\right)$$

**Figure 7:**
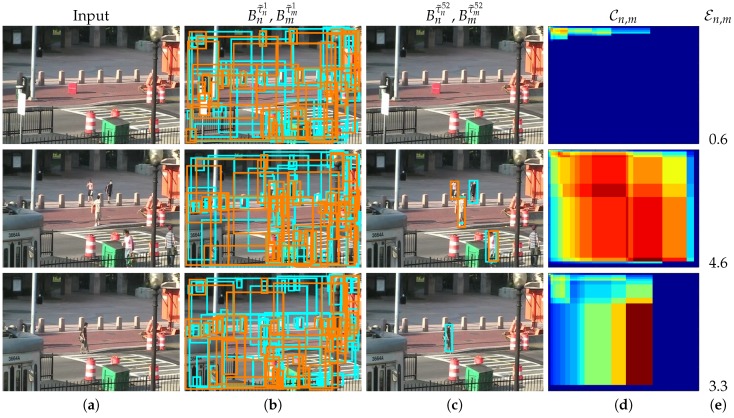
Input images, correlation results, correlation map and entropy results for two Faster R-CNN detectors [1] using VGG (cyan) and ZF models (orange). Three examples (rows) are shown where the column (**a**) are the input images, the next two columns are the obtained bounding boxes for thresholds: (**b**) ${\tilde{\tau }}_{1}^{1}=0$; and (**c**) ${\tilde{\tau }}_{1}^{52}=0.85$ from the full set ${\left\{{\tilde{\tau }}_{n}^{j}\right\}}_{j=1}^{j=L=60}$; (**d**) the correlation map $C_{1,2}$ with color code of (blue) $0\leq C_{1,2}\leq 1$ (red); and (**e**) the corresponding entropy value $E_{1,2}$.

Figure 7 shows three different examples (rows) of correlation maps $C_{n,m}$, the output of two detectors for two different threshold values (low and high thresholds) and the corresponding entropy $E_{n,m}$ values. Note the three different correlation behaviors: the first example shows an empty scene, almost zero FScore similarity for any possible pair-wise correlation and therefore a low entropy value ($E_{n,m}=0.6$); the second example shows an scene with five pedestrians, high FScore similarity for a range of pair-wise correlations and therefore a high entropy value ($E_{n,m}=4.6$); and the third example shows only one person, a medium-high FScore similarity for a range of pair-wise correlations and therefore a medium-high entropy value ($E_{n,m}=3.3$).

Up to this point, we have a set of hypothesis for presence of people obtained for each compared pair of detectors $E_{n,m}$ (i.e., $D_{n}$ and $D_{m}$), which are combined to obtain a final decision (*decision fusion* in Figure 6). Such hypotheses combination is performed as a traditional mixture of experts via weighted voting [36]:

(6) $$E=\sum_{m=1}^{N}\omega^{n,m}\cdot E_{n,m}\;\left(n\neq m\right),$$

where $\omega^{n,m}\in \left[0,1\right]$ is the weight for the hypothesis $E_{n,m}$ achieved by comparing $D_{n}$ and $D_{m}$ and $\sum_{m=1}^{N}\omega^{n,m}=1\;\left(n\neq m\right)$. Although such ensemble voting may benefit from a previous learning stage [37], currently we assume no prior knowledge about detectors performance so we consider equal weighting $\omega^{n,m}=\frac{1}{N-1}$.

In the case of absence of people (i.e., low value of E), we assume the detections outputs are empty (i.e., $B_{n}^{{\tilde{\tau }}_{n}^{i}}=B_{m}^{{\tilde{\tau }}_{m}^{j}}=\emptyset$) and therefore the final configuration for each detector is $\tau_{1}^{*}=\tau_{n}^{*}\ldots =\tau_{N}^{*}=\infty$. This decision has the potential benefit of avoiding any possible false detection but also the possible disadvantage of losing any correct detections (see visual examples in Figure 1). On the other side, in the case of presence of people (i.e., high value of E), a further adaptation process is required, therefore it is necessary to analyze the fine similarity for the adaptation process.

We formulate the detection of frames containing people (i.e., coarse adaptation) as a two-class classification problem where class $q_{1}$ indicates the absence of people in a frame and $q_{2}$ is the opposite class. We classify the frame based on the evidence provided by the entropy E, we evaluate the posterior probability of each class $P(q_{i}|E)$ and we choose the class with largest $P(q_{i}|E)$, i.e., $P\left(q_{1}|E\right)\overset{\omega_{1}}{\underset{\omega_{2}}{≷}}P\left(q_{2}|E\right).$ Then, applying the Bayes Rule results in:

(7) $$\frac{P\left(E|q_{1}\right)P\left(q_{1}\right)}{P(E)}\overset{q_{1}}{\underset{q_{2}}{≷}}\frac{P\left(E|q_{2}\right)P\left(q_{2}\right)}{P(E)}$$

P(E) does not affect the decision rule so it can be eliminated. We simplify to the likelihood ratio ∧(E):

(8) $$\wedge \left(E\right)=\frac{P(E|q_{1})}{P(E|q_{2})}\overset{q_{1}}{\underset{q_{2}}{≷}}\frac{P(q_{2})}{P(q_{1})}$$

Finally, assuming equal priors (absence/presence of people), the decision rule is known as the Likelihood Ratio Test (LRT):

(9) $$\wedge \left(E\right)=\frac{P(E|q_{1})}{P(E|q_{2})}\overset{q_{1}}{\underset{q_{2}}{≷}}1,$$

which in essence turns into finding the first entropy value E that determines the condition $\frac{P(E|q_{1})}{P(E|q_{2})}>1$ and using such value as a threshold for the entropy.

### 3.3. Fine Adaptation

The aim of the fine adaptation is to find the configuration with the highest similarity (i.e., highest value in $C_{n,m}$) to select the best detection threshold for each detector ($\tau_{n}^{*}$ and $\tau_{m}^{*}$, respectively). The threshold hypothesis selection requires searching a single maximum value in $C_{n,m}$, which may contain multiple local maxima. The correlation map $C_{n,m}$ is the similarity ρ between the output of each pair of detectors $B_{n}^{{\tilde{\tau }}_{n}^{i}}$ and $B_{m}^{{\tilde{\tau }}_{m}^{j}}$, and the threshold hypothesis selection can derived as:

(10) $$\left\{\tau_{n}^{n,m},\tau_{m}^{n,m}\right\}:\rho \left(B_{n}^{{\tilde{\tau }}_{n}^{n,m}},B_{m}^{{\tilde{\tau }}_{m}^{n,m}}\right)\geq \rho \left(B_{n}^{{\tilde{\tau }}_{n}^{i}},B_{m}^{{\tilde{\tau }}_{m}^{j}}\right),\forall i,j\left(i,j\in \{1,\ldots L\}\right)$$

where ρ(·,·) is defined as Equation (3).

Our problem for finding the optimal global solution can be formulated by following the Maximum Likelihood Estimation (MLE) criterion once computed $C_{n,m}$:

(11) $$\left\{\tau_{n}^{n,m},\tau_{m}^{n,m}\right\}=\underset{{\tilde{\tau }}_{n}^{i},{\tilde{\tau }}_{m}^{j}}{argmax}\left(\rho (B_{n}^{{\tilde{\tau }}_{n}^{i}},B_{m}^{{\tilde{\tau }}_{m}^{j}})\right),\;\left(i,j\in \{1,\ldots L\}\right).$$

To find such maximum value, we propose using a sub-optimal global search solution of the threshold hypothesis selection problem with lower computational cost requirements, i.e. Simulated Annealing (SA) [38]. SA is a probabilistic technique for approximating the global optimum of a given function. For problems where finding an approximate global optimum is more important than finding a precise local optimum in a fixed amount of time, SA may be preferable to other iterative alternatives such as gradient descent [39].

Moreover, we may assume that the probability of selecting a pair of thresholds (i.e., choosing a specific configuration) depends on the pair of detectors compared. For example, some detectors may tend to use thresholds with low values, whereas other detectors may use high values. Therefore, we include a function g(·,·) to model the prior distribution of thresholds which determines the most likely pairs of thresholds given two detectors. It can be defined as follows:

(12) $$\left\{\tau_{n}^{n,m},\tau_{m}^{n,m}\right\}=\underset{{\tilde{\tau }}_{n}^{i},{\tilde{\tau }}_{m}^{j}}{argmax}\left(\rho \left(B_{n}^{{\tilde{\tau }}_{n}^{i}},B_{m}^{{\tilde{\tau }}_{m}^{j}}\right)\cdot g\left(B_{n}^{{\tilde{\tau }}_{n}^{i}},B_{m}^{{\tilde{\tau }}_{m}^{j}}\right)\right),\left(i,j\in \{1,\ldots L\}\right).$$

Since the solution of Equation (11) or Equation (12) may not be unique, we may obtain various maximum values $\tau_{n}^{n,m}$ (see the darkest area in the bottom-left image in Figure 5a) as the detectors are never totally independent. Therefore, we currently propose three alternatives: selecting the mean, minimum or maximum value among those thresholds $\tau_{n}^{n,m}$ maximizing $C_{n,m}$.

**Figure 8:**
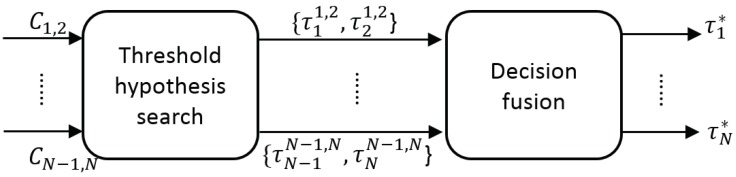
Overview fine adaptation.

After finding the best detection thresholds obtained for each compared pair of detectors $\tau_{n}^{n,m}$ (i.e., $D_{n}$ and $D_{m}$), we combine them to obtain a final configuration for each detector (*decision fusion* in Figure 8).

Such hypotheses combination is performed as in Equation (6) as a traditional mixture of experts via weighted voting as follows:

(13) $$\tau_{n}^{*}=\sum_{m=1}^{N}\omega^{n,m}\cdot \tau_{n}^{n,m}\;\left(n\neq m\right).$$

It is important to note that this equation does not combined people detectors, instead the proposed approach focuses on improving independently each detector by adapting the detection threshold.

## 4. Experimental Results

This section describes the experimental setup to evaluate the proposed coarse-to-fine framework to adapt people detectors during runtime classification, and the results of each part of the framework: coarse adaptation, fine adaptation, and the complete system (see Figure 2).

### 4.1. Setup

We performed the evaluation using the people detection benchmark repository (PDbm (http://www-vpu.eps.uam.es/PDbm/, last accessed December 2018.)) [40]. It has 19 sequences with ground-truth annotations for traditional indoor and outdoor scenarios in computer vision applications: video surveillance, smart cities, etc.

We quantified detection performance for each video frame by precision, recall and FScore metrics [35]. We report the frame-level mean FScore for all tested images as the final performance value. However, to evaluate the impact of the coarse adaptation in the final system, we evaluated the performance in terms of global FScore, i.e., the resulting video-level FScore of the adaptation process for each video and not only frame by frame results.

We applied the adaptation system to six people detectors using publicly available implementations. We used two versions for DPM [32] (Inria and Pascal models), ACF [41] (Inria and Caltech models) and Faster R-CNN [1] (VGG and ZF models).

### 4.2. Coarse Adaptation Results

**Figure 9:**
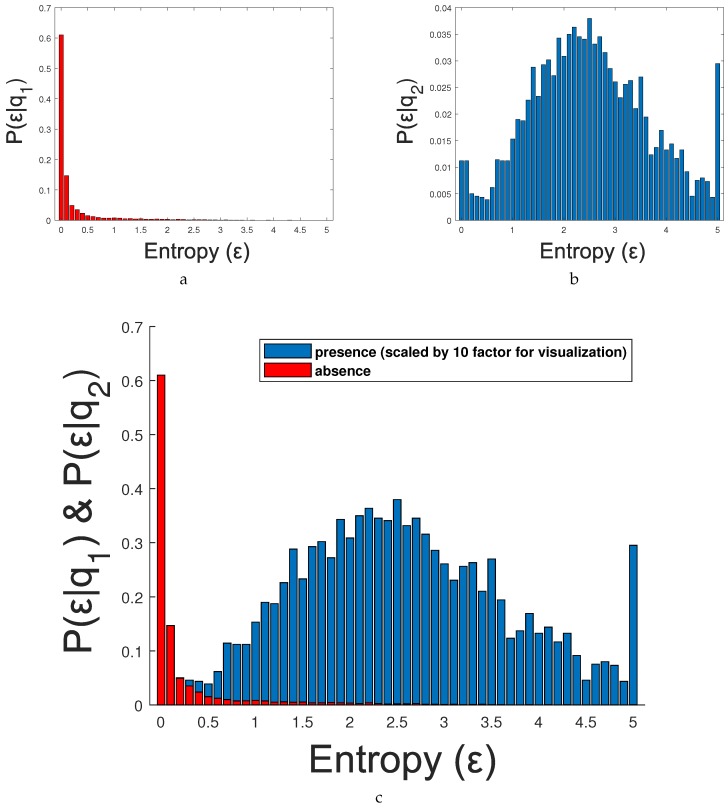
Example of probability density function of absence/presence of people: (**a**) $P(E|q_{1})$; (**b**) $P(E|q_{2})$; and (**c**) both pdfs together. The red distribution correspond to the absence of people (Class 1) and the blue one to the presence of people (Class 2).

We proposed the estimation of the absence/presence of people for each frame, using the entropy of the correlation map $C_{n,m}$ (see Section 3.2). We first estimated the entropy probability density function (pdf) of both classes ($P(E|q_{1})$ and $P(E|q_{2})$) using the training dataset VOC2012 (Visual Object Classes Challenge 2012 [42]). Figure 9a,b shows the estimated entropy pdfs $P(E|q_{1})$ and $P(E|q_{2})$, respectively, while Figure 9c shows both pdfs together. After that, we used the LRT (see Equation (9)) to determine the best entropy threshold between the two classes, i.e., E=0.7.

Then, we validated the absence/presence of people classification approach. We analyzed the results over the evaluation dataset, PDbm [40]. We performed a 10-fold cross-validation evaluation selecting randomly a balanced set of 1000 frames with and without the presence of people. We analyzed the precision (P), recall (R) and FScore (F) for each class (the absence/presence of people, Classes 1 and 2, respectively) and the final FScore sum. Table 2 shows the classification results obtained by a random classifier, by the six detectors independently and by our proposal with different number of thresholds L={5,10,20,40,60}. For the independent detectors, the optimal fix threshold was previously learned with the training dataset VOC2012 (Visual Object Classes Challenge 2012 [42]). The proposed coarse adaptation could classify with around 80% of precision and recall both classes: absence and presence of people. On the other hand, all the other approaches obtained worse results (around 50–60%). The results show clearly how the use of the entropy over the six detectors improve the results significantly in terms of precision, recall and FScore, with respect to the use of the detectors independently and, therefore, versus a random classifier. In addition, the results show how the performance using different number of thresholds L={5,10,20,40,60} are quite homogeneous, getting all of them around 1.6 of FScore sum. For that reason, we use the coarse adaptation with L=5 since it presents a lower computational cost, i.e., lower number of pair-wise correlations between detectors per frame (see detailed analysis in Section 4.3.3 and Table 8).

### 4.3. Fine Adaptation Results

#### 4.3.1. Fine Adaptation: Maximum Likelihood Estimation

We evaluated the fine adaptation stage, Adaptive people Detection by maximizing Correlation (ADC), with five sets with incremental size to test the effect of successively adding detectors to the final result: ADC2 (DPM-I and DPM-P), ADC3 (DPM-I, DPM-P, and ACF-I), ADC4 (DPM-I, DPM-P, ACF-I, and ACF-C), ADC5 (DPM-I, DPM-P, ACF-I, ACF-C, and FRCNN-VGG) and ADC6 (DPM-I, DPM-P, ACF-I, ACF-C, FRCNN-VGG, and FRCNN-ZF).

**Figure 10:**
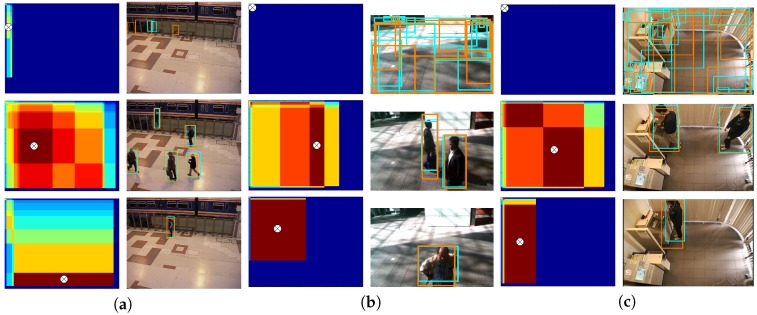
Correlation and threshold selection results between pairs of detectors. Each column pair shows an example of the selected thresholds (cross-marked) in the correlation map (left column) and the corresponding obtained bounding boxes (right column). Column pairs correspond to: (**a**) Faster R-CNN [1] using VGG (cyan) and ZF (orange) models; (**b**) DPM [32] using Inria (cyan) and Pascal (orange) models; and (**c**) ACF [41] using Inria (cyan) and Caltech (orange) models.

Table 3 shows the average results after adapting two and six detectors, ADC2 and ADC6, respectively, with different number of thresholds L={5,10,20,40,60} and strategies to select a threshold $\tau_{n}^{n,m}$ from those values maximizing $C_{n,m}$ (*mean*, *minimum* or *maximum*). In both cases, the results show that the performance increases progressively with the number of thresholds. In addition, the *minimum* strategy obtained in general the worst results and the *mean* strategy obtained slightly better results than the *maximum* one. Figure 10 shows examples of correlation and threshold selection results between pairs of detectors. In the first row, there are three examples of scenes without people and low FScore similarity for any possible pair-wise correlation, while the other two rows include examples from one to five pedestrians and medium-high FScore similarity for a range of pair-wise correlations.

Table 4 shows one example of successively adding detectors to the final configuration from two detectors to six (from ADC2 to ADC6). In general, the results show that the greater is the number of detectors the higher is the performance. For example, the DPM-I increases progressively the performance from 37.1 (ADC2) to 38.2 (ADC6). Aa other examples, the ACF-I increases progressively the performance from 38.3 (ADC3) to 39.5 (ADC6) and the the ACF-C increases progressively the performance from 40.0 (ADC4) to 42.0 (ADC6).

Table 5 shows the comparative results of our approach (ADC6, all six detectors independently of the order or their inclusion) versus two different fixed thresholding approaches ($FT_{PDbm}$ and $FT_{VOC12}$). The $FT_{PDbm}$ approach is the ideal case, the optimal threshold is previously learned with the chosen evaluation dataset (PDbm [40]) and the $FT_{VOC12}$ is a more realistic approach, where the optimal threshold is previously learned with the training dataset VOC2012 (Visual Object Classes Challenge 2012 [42]). The results show clearly that the use of our adaptive threshold approach ADC6 significantly improves the results of any of the individual detectors using a fixed threshold (10.1% and 18.6% average improvement with respect to $FT_{PDbm}$ and $FT_{VOC12}$, respectively).

Additionally, we also evaluate the Fine adaptation stage (ADC6) over a different dataset, the MILAN dataset [43]. This dataset includes eleven challenging, publicly available video sequences with ground truth (TUD-Stadtmitte, TUD-Campus and TUD-Crossing, S1L1 (1 and 2), S1L2 (1 and 2), S2L1, S2L2, S2L3 and S3L1). The first three sequences are recorded in real-world busy streets, the complexity in terms of crowd or occlusions is medium or low (fewer than 10 pedestrians are present simultaneously). The last eight sequences are part of the PETS 2009/2010 benchmark [44]. They are recorded outdoors from an elevated point of view, corresponding to a typical surveillance setup. These scenarios include higher complexity in terms of crowds and occlusions than the previous ones (generally more than 10 pedestrians are present simultaneously).

Table 6 shows the comparative results of our approach (ADC6) versus two different Fixed Thresholding approaches ($FT_{MILAN}$ and $FT_{VOC12}$) over the MILAN dataset [43]. As in the previous experiment, The $FT_{MILAN}$ approach is the ideal case and the $FT_{VOC12}$ is a more realistic approach. The ADC6 presents similar results as with the previous dataset. In this case, the initial or fixed thresholding results are higher, therefore the potential improvement is slightly smaller, even though our adaptive approach ADC6 significantly improves the results of any of the individual detectors using a fixed threshold (8.3% and 12.9% average improvement with respect to $FT_{MILAN}$ and $FT_{VOC12}$, respectively).

#### 4.3.2. Fine Adaptation: Maximum A Posteriori Estimation

As commented in Section 4.3.1, the previous results are for the threshold hypothesis selection using the Maximum Likelihood Estimation (MLE). However, the results can be improved including the prior distributions of any pair of thresholds configurations, i.e., the correlation map $C_{n,m}$. Therefore, we evaluated the results using the Maximum A Posteriori Estimation (MAP). Firstly, during the optimal fix threshold learning for evaluation comparison, we also learned the prior distributions of each pair of detector with the training dataset VOC2012 (Visual Object Classes Challenge 2012 [42]) and then we evaluated the results of our approach ADC6 over PDbm including the estimated posteriori in the threshold hypothesis selection.

**Figure 11:**
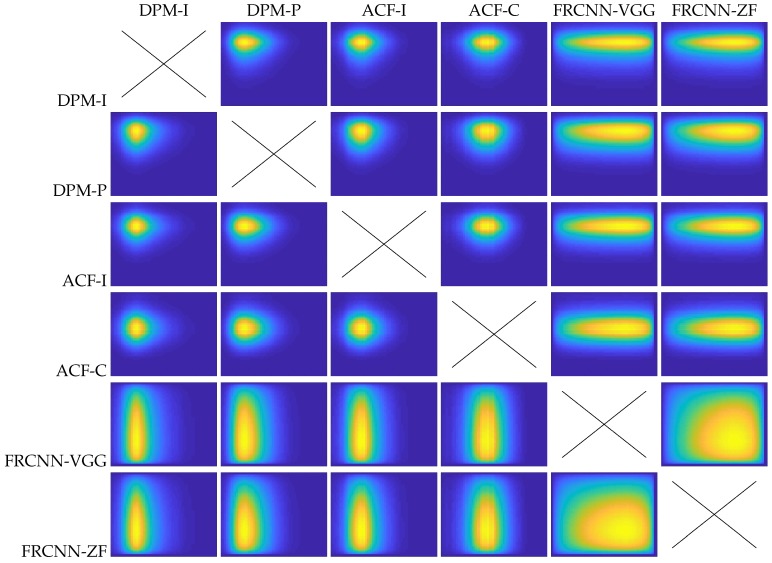
Visual representation of prior distributions of any pair of thresholds configurations.

Figure 11 includes a visual representation of the 15 different prior distributions, one for each pair of six detectors and their 15 mirrored versions. Note the clear different behavior between different detectors. While the DPM and ACF versions present a more concentrated range of best thresholds, both FRCNN variations present a sparser range of best thresholds. It is due to the better detection performance of the FRCNN itself and therefore any possible improvement versus a predefined fix threshold will be more difficult. Table 7 shows the comparative results using the MLE versus using the MAP. The results show clearly that the use of our adaptive threshold approach ADC6 with the MAP improves the results of any of the individual detectors without the MAP (3.3% average improvement).

#### 4.3.3. Fine Adaptation: Threshold Hypothesis Selection

We propose using a sub-optimal global search solution of the threshold hypothesis selection problem with lower computational cost requirements, the Simulated Annealing (SA) [38]. We compared SA against other search alternatives; for example, applying a subset of thresholds $K=\left\lfloor L/k\right\rfloor$ (see Section 3.1.1), being *k* the sub-sampling factor in the decision space, i.e., k∈R and k>1. In particular, we evaluated four sub-sampling factors from the original decision space L=60 (Exhaustive Search, ES), the sub-optimal subsets of thresholds are K={40,20,10,5}. We also evaluated three non-regular sub-sampling patterns, the Three Step Search (TSS) [45], the Four Step Search (FSS) [46], and the Diamond Search (DS) [47]. Finally, we also evaluated two traditional global optimization pattern search approaches: the Pattern/Direct Search (PS) [48] and the Particle Swarm Optimization (PSO) [49].

Table 8 shows the comparative results in terms of FScore and computational cost (number and percentage of operations per each frame), between different threshold hypothesis selection approaches, including regular sub-sampling patterns with sub-optimal subsets of thresholds K={40,20,10,5}, non-regular sub-sampling patterns (TSS, FSS and DS) and more traditional global optimization approaches (PS, PSO, and SA). The results show clearly how the exhaustive approach, i.e., searching in the original decision space L=60, obtains the best results but the highest computational cost. Logically, any sub-optimal global search solution of the threshold hypothesis selection problem will obtain worse results in terms of FScore, but also a reduction of the computational cost. The use of different sub-optimal subsets of thresholds (K={40,20,10,5}), obtained progressively worse FScore results (from 42.5 to 37.4 respectively) but with a strong reduction in terms of percentage of operations (from 44.4% to 0.7%, respectively, being the 100% of operations per each frame required with K=60). The use of non-regular sub-samplings also obtained worse FScore results (between 32.8 and 39.9) but with always a drastic reduction in terms of percentage of operations (only between 0.4% and 1.1% of operations per each frame are required). In particular, FSS obtains the best ratio between FScore results and computational cost. Finally, the use of more traditional global optimization pattern search also obtained worse FScore results (between 35.7 and 42.0) with a drastically reduction in terms of percentage of operations only between 0.2% and 5.0% of operations per each frame are required). In particular, SA obtained the best FScore results (42.0) but also a strong computational cost reduction in terms of percentage of operations (only 5.0% of operations per each frame are required). Note the progressive reduction of FScore and computational cost of the sub-optimal subsets of threshold (K={40,20,10,5}), the significant reduction of FScore with the use of any non-regular sub-samplings (TSS, FSS and DS) but with a strong computational cost reduction, and the different behaviors of the three more traditional global optimization pattern search, being significantly better the use of SA.

### 4.4. Final Adaptation System (Coarse and Fine)

We evaluated the whole proposed framework (coarse and fine adaptation), described in Section 3. The coarse and fine adaptation were evaluated at frame-level, as shown, respectively, in Section 4.2 and Section 4.3. In particular, we evaluated the use of our coarse analysis to identify the representative frames for a possible adaptation of the system; those frames without the presence of people were discarded and those with the presence of people were further analyzed locally. To evaluate the whole coarse-to-fine adaptation process, we compared the results without and with the inclusion of the coarse adaptation stage at video-level. The system without the coarse adaptation corresponds to the proposed fine adaptation ADC6 with MLE or MAP, as evaluated in detail in, respectively, Section 4.3.1 and Section 4.3.2. We defined the entropy coarse adaptation threshold with L=5 and according to the Likelihood Ratio Test, i.e., E=0.7 (see detailed reasoning in Section 4.2). Generally, the inclusion of the coarse adaptation obtained worse results in terms of the number of true positive detections because those frames misclassified as if there were no people certainly produce missed detections. However, the coarse adaptation also obtained better results in terms of false positive detections, since those frames correctly classified as if there is no people potentially reduce the total number of false detections (see Section 4.2 for further details). In addition, the inclusion of the coarse adaptation significantly reduces the computational cost since the fine adaptation in every frame demands a higher computational cost.

Table 9 shows the final adaptation system results for each detection algorithm, with the use of MLE or MAP. In general, the use of the coarse adaptation introduces a significant improvement in the evaluation results (between 21.7% and 90.8% of improvement). It is due to the balance between the number of the false detections and the true positive detections.

Table 10 shows the comparative results in terms of FScore and computational cost (number and percentage of operations per each frame), between the use of a fixed threshold $FT_{VOC12}$ and the final adaptation system results (MLE or MAP). There is also an improvement in FScore performance (10.8% and 16.1% average improvement with respect to the fixed thresholding approach $FT_{VOC12}$, MLE and MAP, respectively) and almost a 50% of reduction in terms of computational cost per frame.

**Figure 12:**
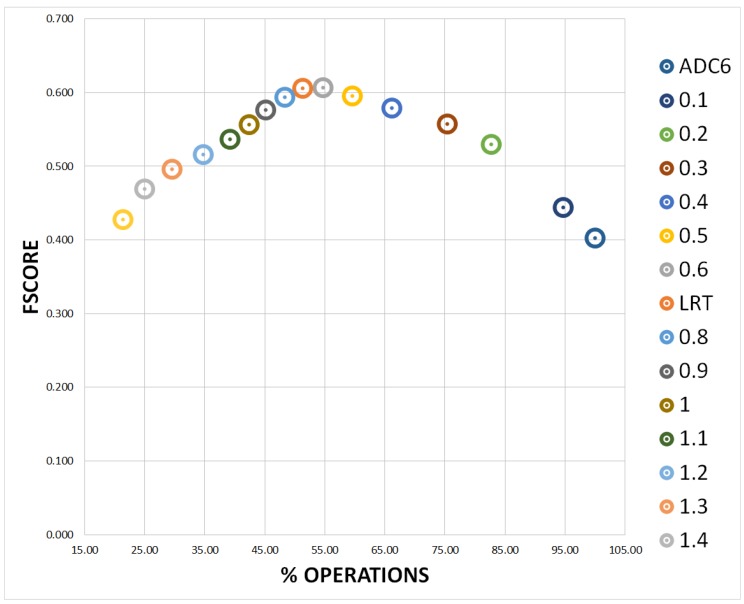
Comparative video analysis results with different coarse adaptation configurations, absence/presence of people classification decision, from entropy E=0 to 1.5. Global FScore results for each video versus computational cost in terms of percentage of operations.

To understand the relation between the entropy coarse adaptation threshold (E) and the performance in terms of FScore and computational cost, we analyzed the performance of our final system with MLE (MAP version present the exactly same behavior) for different entropy coarse adaptation thresholds, E=0,0.1,…,1.5. Note that E=0 corresponds to the absence of coarse adaptation, only fine adaptation, i.e., ADC6. Figure 12 shows the final results versus the corresponding computational cost in terms of percentage of operations. Note clearly the progressive increase in terms of FScore from entropy E=0 until the LRT (E=0.7) and the posterior reduction in terms of FScore until E=1.5. In general, avoiding frames without the presence of people improves the results avoiding false detections until the LRT (E=0.7), after this point the balance between the false detections and the missed detections starts decreasing the performance.

## 5. Conclusions

We have presented a coarse-to-fine framework to automatically adapt people detectors during runtime classification. This proposal explores multiple thresholding hypotheses and exploits the correlation among pairs of detector outputs to determine the best configuration. The coarse adaptation determines the presence/absence of people in every frame and therefore the necessity/not necessity of adaptation of the system. The fine adaptation obtains the optimal detection threshold for each detector in every frame. The proposed approach uses standard state-of-the-art detector outputs (bounding boxes), therefore it can employ various types of detectors. This framework allows the automatic threshold adaptation without requiring a re-training process and therefore without requiring any additional manually labeled ground truth apart from the offline training of the detection model.

The proposed coarse adaptation is able to classify with around 80% of precision and recall both classes absence and presence of people. The fine adaptation results (both MLE and MAP versions) demonstrate that any correlation up to six detectors outperforms state-of-the-art detectors, whose thresholds are optimally trained in advance. In addition, we also explored other sub-optimal threshold hypothesis selection approaches with lower computational cost requirements (number of pair-wise correlations between detectors per frame). In particular, the SA search obtains almost the exhaustive FScore results but with a drastic computational cost reduction. Overall, the final coarse-to-fine framework also outperforms state-of-the-art detectors, for both frame by frame and video analysis results, with a computational cost reduction of around 50%.

For future work, we will study other threshold selection and fusion alternatives and we will apply this proposal to other detectors and object types. We will also explore other additional configurations and not only the detection threshold, for example the position of the bonding box, scale of the detected objects, pose, etc.

We acknowledge that running six detectors significantly increases the required resources as compared to running a single detector. However, this adaptation scheme may not need to be applied for each frame of a video sequence and it may be used periodically (e.g., every 1 or 5 s) or be used on-demand (e.g., when scene conditions change after a camera moves). In this case, the computational cost is considerably decreased as we may not apply our adaptation to each frame. We will consider such applicability in real systems as future work.

## Figures and Tables

**Table 1:** Comparison between the main reviewed approaches for adapting people detectors. FB, feature-based approaches; MD, model-based approaches; PBD, part-based detector; I, image; V, video.

Ref.	Type	Strategy	Target Domain			Online Fashion	Adapted Parameters	Comments
			Re-Training	Labels	Data			
[17]	FB	-	Yes	Yes	I	No	New classifier	Full learning of grid-classifiers
[10]	FB	Transfer learning	Yes	Yes	I	No	Tuned classifier	Data augmentation for CNNs
[18]	FB	Transfer learning	Yes	Yes	I	No	Tuned classifier	Feature selection for attributes
[19]	FB	Transfer learning	Yes	No	I	No	Tuned classifier	Sample selection using super-pixels
[3]	FB	Transfer learning	Yes	No	I	No	Tuned classifier	Sample selection using confidence scores
[20]	FB	Transfer learning	Yes	No	I	No	Tuned classifier	Sample selection and propagation
[4]	FB	Transfer learning	Yes	No	I	No	Tuned classifier	Sample selection using multiple cues
[21]	FB	Transfer learning	Yes	No	I	Yes	Tuned classifier	Added losses at image and instance level
[22]	FB	Transfer learning	Yes	Yes	I	No	Tuned classifier	Use of synthetic data
[5]	MB	-	No	No	I	Yes	Prior probabilities	Multi-class Bayesian classifier
[26]	MB	Context	No	No	I	Yes	PBD configurations	Requires spatial context of scene
[27]	MB	Context	Yes	No	I	No	Prior probabilities	Requires knowledge rules of scene
[11]	MB	Model combination	No	No	I	Yes	Feature weighting	Combination of multiple modalities
[28]	MB	Model selection	No	No	I	Yes	Adaptive selection	Visible or non-visible light images
[7]	MB	Detector ranking	No	No	I	Yes	Best selection from pool	Source-target domain similaritiy
[13]	MB	Detection-tracking	Yes	No	I	Yes	Tuned classifier	Sample selection by tracking
**Proposed**	**MB**	**Cross correlation**	**No**	**No**	I/V	**Yes**	**Detection threshold**	**Maximization mutual information**

**Table 2:** Absence/presence of people classification results obtained by a random classifier, by the six detectors independently and using our proposal with different number of thresholds L={5,10,20,40,60}.

Approach	P1	R1	P2	R2	F1	F2	F1 + F2
Random	0.50	0.40	0.50	0.60	0.48	0.57	1.01
DPM-I	0.55	0.47	0.54	0.61	0.49	0.56	1.01
DPM-P	0.49	0.67	0.48	0.30	0.56	0.39	0.95
ACF-I	0.52	0.50	0.52	0.54	0.52	0.51	1.03
ACF-C	0.53	0.40	0.52	0.64	0.46	0.57	1.03
FRCNN-VGG	0.54	0.36	0.52	0.69	0.44	0.59	1.03
FRCNN-ZF	0.51	0.27	0.50	0.74	0.36	0.60	0.95
**Proposed** L=5	0.88	0.71	0.76	0.91	0.79	0.83	1.62
**Proposed** L=10	0.84	0.78	0.80	0.85	0.77	0.80	1.57
**Proposed** L=20	0.82	0.81	0.81	0.82	0.77	0.79	1.56
**Proposed** L=40	0.81	0.82	0.82	0.81	0.77	0.79	1.56
**Proposed** L=60	0.81	0.83	0.83	0.80	0.78	0.79	1.57

**Table 3:** Average FScore of adapted detectors for different strategies to select a threshold $\tau_{n}^{n,m}$ from those values maximizing $C_{n,m}$ obtained with various threshold with L=5,10,20,40 and 60. Bold indicates best result for: (a) ADC2; and (b) ADC6. Data adapted from [15].

	Strategy	Mean	Minimum	Maximum
(a) ADC2	L=5	33.2	33.4	33.2
	L=10	35.1	34.9	35.0
	L=20	35.9	35.7	35.7
	L=40	**36.3**	35.9	36.0
	L=60	**36.3**	35.9	36.0
(b) ADC6	L=5	39.8	38.8	40.1
	L=10	41.6	39.9	41.7
	L=20	42.4	39.6	42.2
	L=40	**42.7**	39.0	42.2
	L=60	**42.7**	39.1	42.0

**Table 4:** Average FScore of the five ADC combinations from ADC2 to ADC6. Percentage increase (%Δ) calculated for each detector with respect to the previously obtained performance just before the additional detector inclusion in the combination (in bold), from ADC2 to ADC5, respectively. Data adapted from [15].

	ADC Combinations								
	ADC2	ADC3	%Δ	ADC4	%Δ	ADC5	%Δ	ADC6	%Δ
FRCNN-ZF [1]	-	-	-	-	-	-	-	47.2	-
FRCNN-VGG [1]	-	-	-	-	-	**51.6**	-	51.8	0.4
ACF-C [41]	-	-	-	**40.0**	-	41.6	4.0	42.0	5.0
ACF-I [41]	-	**38.3**	-	38.6	0.8	39.3	2.6	39.5	3.1
DPM-P [32]	**35.3**	35.9	1.7	36.2	2.5	36.9	4.5	37.0	4.8
DPM-I [32]	**37.1**	37.2	0.3	37.6	1.3	38.2	3.0	38.2	3.0

**Table 5:** Comparison in terms of average FScore between two fixed thresholding approaches and the ADC6 over PDbm dataset. Percentage increase (%Δ^PDbm^ and %Δ^VOC12^) calculated with respect to the fixed thresholding approaches, $FT_{PDbm}$ and $FT_{VOC12}$, respectively.

	Fixed Threshold		Proposed Threshold Adaptation		
	${\mathrm{FT}}_{\mathrm{PDbm}}$	${\mathrm{FT}}_{\mathrm{VOC}12}$	ADC6	%Δ^PDbm^	%Δ^VOC12^
DPM-I [32]	33.9	29.9	38.2	12.7	27.8
DPM-P [32]	32.9	31.3	37.0	12.5	18.2
ACF-I [41]	35.2	32.1	39.5	12.2	23.1
ACF-C [41]	36.6	35.2	42.0	14.8	19.3
FRCNN-VGG [1]	50.1	46.0	51.8	3.4	12.6
FRCNN-ZF [1]	44.2	41.2	47.2	6.8	14.6
Average	38.8	36.0	42.7	10.1	18.6

**Table 6:** Comparison in terms of average FScore between two fixed thresholding approaches and the ADC6 over MILAN dataset. Percentage increase (%Δ^MILAN^ and %Δ^VOC12^) calculated with respect to the fixed thresholding approaches, $FT_{MILAN}$ and $FT_{VOC12}$, respectively.

	Fixed Threshold		Proposed Threshold Adaptation		
	${\mathrm{FT}}_{\mathrm{MILAN}}$	${\mathrm{FT}}_{\mathrm{VOC}12}$	ADC6	%Δ^MILAN^	%Δ^VOC12^
DPM-I [32]	50.1	47.1	54.5	8.8	15.7
DPM-P [32]	54.5	52.5	59.1	8.5	12.7
ACF-I [41]	65.4	61.4	67.8	3.7	10.4
ACF-C [41]	64.8	61.8	69.4	7.1	12.3
FRCNN-VGG [1]	70.1	66.1	76.6	9.3	16.0
FRCNN-ZF [1]	65.3	61.3	73.4	12.5	19.8
Average	61.7	59.2	66.8	8.3	12.9

**Table 7:** ADC6 FScore results including the maximum a posteriori estimation in the threshold hypothesis selection or fine adaptation.

	Proposed Threshold Adaptation		
	ADC6 (MLE)	ADC6 (MAP)	%Δ
DPM-I [32]	38.2	40.2	5.2
DPM-P [32]	37.0	38.4	3.8
ACF-I [41]	39.5	40.7	3.0
ACF-C [41]	42.0	43.9	4.5
FRCNN-VGG [1]	51.8	52.7	1.7
FRCNN-ZF [1]	47.2	48.9	3.6
Average	42.7	44.1	3.3

**Table 8:** Comparative results between different search approaches for threshold hypothesis selection, including regular sub-sampling patterns with sub-optimal subsets of thresholds K={40,20,10,5}, non-regular sub-sampling patterns (TSS, FSS and DS) and more traditional global optimization approaches (PS, PSO, SA). Results in terms of FScore and computational cost (number and percentage of operations per each frame).

Search	FScore	#Operations	%Operations
ES (*K* = 60)	42.6	54,000	100
*K* = 40	42.5	24,000	44.4
*K* = 20	41.9	6000	11.1
*K* = 10	40.7	1500	2.8
*K* = 5	37.4	375	0.7
FSS [46]	39.9	285	0.5
TSS [45]	37.5	615	1.1
DS [47]	32.8	240	0.4
PS [48]	35.7	83	0.2
PSO [49]	41.8	489	0.9
SA [38]	42.0	1270	5.3

**Table 9:** Final adaptation system average results with likelihood ratio test (E=0.7). Video-level evaluation results.

	Final Adaptation System					
	MLE			MAP		
	ADC6	Final	%Δ	ADC6	Final	%Δ
DPM-I [32]	37.3	53.9	+44.5	43.9	57.8	+31.7
DPM-P [32]	36.5	54.5	+49.3	37.1	57.6	+55.3
ACF-I [41]	45.4	57.9	+27.5	49.8	60.6	+21.7
ACF-C [41]	40.0	55.4	+38.5	44.6	59.9	+34.3
FRCNN-VGG [1]	38.8	73.8	+90.2	39.3	75.0	+90.8
FRCNN-ZF [1]	43.8	67.9	+55.0	45.2	70.2	+55.3

**Table 10:** Final adaptation system for each video average results.

	Video Results		
**Search**	**FScore**	**#Operations**	**%Operations**
$FT_{VOC12}$	54.7	-	-
ADC6 (MLE)	40.3	2.73 × 10^9^	100.0
Final (MLE)	60.6	1.40 × 10^9^	51.3
%Δ^ADC6^	+50.4	-	-
%Δ^VOC12^	+10.8	-	-
ADC6 (MAP)	43.3	2.73 × 10^9^	100.0
Final (MAP)	63.5	1.40 × 10^9^	51.3
%Δ^ADC6 (MAP)^	+46.7	-	-
%Δ^VOC12^	+16.1	-	-

## References

[1] Ren S., He K., Girshick R., Sun J. (2017). Faster R-CNN: Towards Real-Time Object Detection with Region Proposal Networks. IEEE Trans. Pattern Anal. Mach. Intell..

[2] Redmon J., Farhadi A. YOLO9000: Better, Faster, Stronger. Proceedings of the IEEE Conference on Computer Vision and Pattern Recognition (CVPR).

[3] Xingyu Z., Wanli O., Meng W., Xiaogang W. Deep Learning of Scene-Specific Classifier for Pedestrian Detection. Proceedings of the European Conference on Computer Vision (ECCV).

[4] Wang X., Wang M., Li W. (2014). Scene-Specific Pedestrian Detection for Static Video Surveillance. IEEE Trans. Pattern Anal. Mach. Intell..

[5] Royer A., Lampert C.H. Classifier adaptation at prediction time. Proceedings of the IEEE Conference on Computer Vision and Pattern Recognition (CVPR).

[6] Kalinke T., Tzomakas C., Seelen W.V. A Texture-based Object Detection and an adaptive Model-based Classification. Proceedings of the IEEE Intelligent Vehicles Symposium.

[7] Zhang S., Zhu Q., Roy-Chowdhury A. Adaptive algorithm selection, with applications in pedestrian detection. Proceedings of the IEEE International Conference on Image Processing (ICIP).

[8] Karaoglu S., Liu Y., Gevers T. (2016). Detect2Rank: Combining Object Detectors Using Learning to Rank. IEEE Trans. Image Process..

[9] Htike K.K., Hogg D. (2016). Adapting pedestrian detectors to new domains: A comprehensive review. Eng. Appl. Artif. Intell..

[10] Dimou A., Alvarez F. Multi-target detection in CCTV footage for tracking applications using deep learning techniques. Proceedings of the IEEE International Conference on Image Processing (ICIP).

[11] Mees O., Eitel A., Burgard W. Choosing Smartly: Adaptive Multimodal Fusion for Object Detection in Changing Environments. Proceedings of the IEEE/RSJ International Conference on Intelligent Robots and Systems (IROS).

[12] Verma A., Hebbalaguppe R., Vig L., Kumar S., Hassan E. Pedestrian Detection via Mixture of CNN Experts and Thresholded Aggregated Channel Features. Proceedings of the IEEE International Conference on Computer Vision (ICCV).

[13] Kalal Z., Mikolajczyk K., Matas J. (2012). Tracking-Learning-Detection. IEEE Trans. Pattern Anal. Mach. Intell..

[14] Gaidon A., Zen G., Rodriguez J. Self-Learning Camera: Autonomous Adaption of Object Detectors to Unlabeled Video Streams. Proceedings of the European Conference on Computer Vision (ECCV).

[15] Garcia-Martin A., SanMiguel J.C. Adaptive people detection based on cross-correlation maximization. Proceedings of the IEEE International Conference on Image Processing (ICIP).

[16] Xu J., Ramos S., Vazquez D., Lopez A.M. (2014). Domain Adaptation of Deformable Part-Based Models. IEEE Trans. Pattern Anal. Mach. Intell..

[17] Roth P.M., Sternig S., Grabner H., Bischof H. Classifier grids for robust adaptive object detection. Proceedings of the IEEE Conference on Computer Vision and Pattern Recognition (CVPR).

[18] Liu S., Kovashka A. Adapting attributes by selecting features similar across domains. Proceedings of the IEEE Winter Conference on Applications of Computer Vision (WACV).

[19] Shu G., Dehghan A., Shah M. Improving an object detector and extracting regions using superpixels. Proceedings of the IEEE Conference on Computer Vision and Pattern Recognition (CVPR).

[20] Ye Q., Zhang T., Ke W., Qiu Q., Chen J., Sapiro G., Zhang B. Self-Learning Scene-Specific Pedestrian Detectors Using a Progressive Latent Model. Proceedings of the IEEE Conference on Computer Vision and Pattern Recognition (CVPR).

[21] Chen Y., Li W., Sakaridis C., Dai D., Van Gool L. Domain Adaptive Faster R-CNN for Object Detection in the Wild. Proceedings of the IEEE Conference on Computer Vision and Pattern Recognition (CVPR).

[22] Hattori H., Boddeti V.N., Kitani K., Kanade T. Learning scene-specific pedestrian detectors without real data. Proceedings of the IEEE Conference on Computer Vision and Pattern Recognition (CVPR).

[23] Vazquez D., Lopez A.M., Marin J., Ponsa D., Geronimo D. (2014). Virtual and real world adaptationfor pedestrian detection. IEEE Trans. Pattern Anal. Mach. Intell..

[24] Garcia-Martin A., Martinez J.M. (2012). On collaborative people detection and tracking in complex scenarios. Image Vis. Comput..

[25] Espinace P., Kollar T., Roy N., Soto A. (2013). Indoor scene recognition by a mobile robot through adaptive object detection. Robot. Auton. Syst..

[26] Garcia-Martin A., SanMiguel J.C. (2015). Context-aware part-based people detection for video monitoring. Electron. Lett..

[27] Singh K.K., Divvala S., Farhadi A., Lee Y.J. DOCK: Detecting Objects by transferring Common-sense Knowledge. Proceedings of the European Conference on Computer Vision (ECCV).

[28] Kang J.K., Hong H.G., Park K.R. (2017). Pedestrian Detection Based on Adaptive Selection of Visible Light or Far-Infrared Light Camera Image by Fuzzy Inference System and Convolutional Neural Network-Based Verification. Sensors.

[29] Sangineto E., Fleet D., Pajdla T., Schiele B., Tuytelaars T. (2014). Statistical and Spatial Consensus Collection for Detector Adaptation. Computer Vision—ECCV 2014: 13th European Conference, Zurich, Switzerland, 6–12 September 2014, Proceedings, Part III.

[30] Conaire C.O., O’Connor N.E., Smeaton A.F. Detector adaptation by maximising agreement between independent data sources. Proceedings of the IEEE Conference on Computer Vision and Pattern Recognition (CVPR).

[31] SanMiguel J.C., Suja S. (2013). Skin detection by dual maximization of detectors agreement for video monitoring. Pattern Recognit. Lett..

[32] Felzenszwalb P., Girshick R.B., McAllester D., Ramanan D. (2010). Object Detection with Discriminatively Trained Part-Based Models. IEEE Trans. Pattern Anal. Mach. Intell..

[33] Garcia-Martin A., Martinez J.M. (2015). Post-processing approaches for improving people detection performance. Comput. Vis. Image Underst..

[34] Leibe B., Seemann E., Schiele B. Pedestrian Detection in Crowded Scenes. Proceedings of the IEEE Conference on Computer Vision and Pattern Recognition (CVPR).

[35] Garcia-Martin A., Martinez J.M. (2015). People detection in surveillance: Classification and evaluation. IET Comput. Vis..

[36] Ionescu B., Benois-Pineau J., Piatrik T., Quenot G. (2014). Fusion in Computer Vision: Understanding Complex Visual Content.

[37] Baruque B., Corchado E. (2011). Fusion Methods for Unsupervised Learning Ensembles.

[38] Kirkpatrick S., Gelatt C.D., Vecchi2 M.P. (1983). Optimization by Simulated Annealing. Science.

[39] Goldstein A.A. (1962). Cauchy’s method of minimization. Numer. Math..

[40] Garcia-Martin A., Alcedo B., Martinez J.M. (2015). PDbm: People detection benchmark repository. Electron. Lett..

[41] Dollar P., Appel R., Belongie S., Perona P. (2014). Fast Feature Pyramids for Object Detection. IEEE Trans. Pattern Anal. Mach. Intell..

[42] Everingham M., Van Gool L., Williams C.K.I., Winn J., Zisserman A. The PASCAL Visual Object Classes Challenge 2012 (VOC2012) Results. http://host.robots.ox.ac.uk/pascal/VOC/voc2012/.

[43] Milan A., Roth S., Schindler K. (2014). Continuous Energy Minimization for Multitarget Tracking. IEEE Trans. Pattern Anal. Mach. Intell..

[44] PETS International Workshop on Performance Evaluation of Tracking and Surveillance. http://www.cvg.reading.ac.uk/PETS2009/a.html.

[45] Li R., Zeng B., Liou M.L. (1994). A new three-step search algorithm for block motion estimation. IEEE Trans. Circuits Syst. Video Technol..

[46] Lai-Man P., Wing-Chung M. (1996). A novel four-step search algorithm for fast block motion estimation. IEEE Trans. Circuits Syst. Video Technol..

[47] Zhu S., Ma K.K. (2000). A new diamond search algorithm for fast block-matching motion estimation. IEEE Trans. Image Process..

[48] Hooke R., Jeeves T.A. (1961). “ Direct Search” Solution of Numerical and Statistical Problems. J. ACM.

[49] Kennedy J., Eberhart R. Particle swarm optimization. Proceedings of the IEEE International Conference on Neural Networks.

